# Effects of replacing soybean meal with cottonseed meal in amino acid balanced diets on growth performance, apparent digestibility, ruminal fermentation, and microbial diversity in fattening Dorper × Hu crossbred sheep

**DOI:** 10.3389/fvets.2025.1681407

**Published:** 2025-11-06

**Authors:** Yuanrui Yue, Jianwei Lin, Gang Lv, Baocang Liu, Xiaoyu Deng, Yunjie Li, Xiaobin Li, Kaixu Chen

**Affiliations:** 1 Xinjiang Key Laboratory of Herbivore Nutrition for Meat and Milk, College of Animal Science, Xinjiang Agricultural University, Urumqi, China; 2 Xinjiang Taikun Group Co., Ltd., Changji, China

**Keywords:** cottonseed meal, amino acid balance, growth performance, blood biochemical indices, apparent digestibility, ruminal microbial diversity

## Abstract

Amino acid balanced cottonseed meal can effectively replace soybean meal in sheep diets, improving energy utilization without harming growth or health. This study aimed to investigate the effects of replacing soybean meal with cottonseed meal under amino acid balance on growth performance, blood biochemical indices, apparent digestibility, antioxidant function, and ruminal microbial diversity in fattening Dorper × Hu crossbred sheep. A total of 112 healthy 90-day-old Dorper × Hu crossbred rams with similar body weights were randomly divided into 4 groups with 28 sheep each, for a 63-day trial. The control group (CON) was fed a corn-soybean meal diet; T1 group replaced 50% soybean meal with cottonseed meal and balanced lysine and methionine; T2 group replaced 100% soybean meal with cottonseed meal and balanced lysine and methionine; T3 group replaced 100% soybean meal with degossypolized cottonseed meal and balanced lysine and methionine. Results showed no significant differences among groups in average daily gain, average daily dry matter intake, feed conversion ratio, blood biochemical indices, antioxidant indices, and ruminal microbial *α* and *β* diversity (*p* > 0.05). Cottonseed meal replacement significantly affected apparent digestibility, with T2 group exhibiting the highest digestibility of crude protein, crude fat, neutral detergent fiber, and acid detergent fiber (*p* < 0.05). Ruminal propionic acid concentration was significantly higher in replacement groups than in CON (*p* < 0.01), and acetic acid-to-propionic acid ratio was lower (*p* < 0.05). LEfSe analysis revealed the most microbial biomarkers enriched in T3 group. In conclusion, under amino acid balance, cottonseed meal can fully replace soybean meal without compromising growth and health while improving nutrient digestibility and rumen fermentation. These findings provide a scientific basis for soybean meal reduction strategies in ruminant feeding. Future research should explore optimal substitution strategies under different production systems and investigate long-term effects on meat quality and economic benefits.

## 1 Introduction

In livestock production, protein constitutes an essential nutrient critical for animal growth and development. The quality of protein feed directly impacts animal health status and productivity levels. Soybean meal, as one of the most prevalent plant protein sources, is widely recognized as a high-quality plant protein ingredient due to its high protein content, comprehensive essential amino acid profile, and superior digestibility and utilization rates. However, in recent years, the shortage of high-quality protein feed ingredients has emerged as a critical constraint limiting sustainable development in animal husbandry. To optimize protein nutrition supply structures while ensuring animal production efficiency and welfare, there is an urgent need to develop alternative high-quality protein feed resources to effectively alleviate the protein source insufficiency challenges facing the livestock industry (1, 2).

China represents the world’s largest cotton consumer and second-largest cotton producer, generating substantial quantities of cottonseed meal annually. According to the National Bureau of Statistics’ 2024 announcement regarding cotton production, national cotton output totaled 6.164 million tons. Based on calculations where cottonseed comprises approximately 1.5 times the total cotton weight, cottonseed production exceeded 9 million tons. Notably, Xinjiang region accounts for 92.2% of national production while representing 22% of global cotton output (3, 4). Cottonseed meal constitutes a byproduct derived from cottonseed following delinting, dehulling, and oil extraction processes. According to the Chinese Feed Composition and Nutritional Value Tables, its crude protein content reaches 47% (5). However, compared to soybean meal, cottonseed meal contains multiple antinutritional factors including free gossypol, crude fiber, and phytic acid. These compounds potentially exert deleterious effects on digestive systems, growth performance, and organ functionality in animals, thereby restricting its widespread application in animal production systems (6, 7). Recent investigations by Gu et al. demonstrated that dietary substitution of soybean meal with fermented cottonseed meal effectively ameliorated intestinal microbial community structure in piglets, subsequently enhancing immunological function, antioxidant capacity, and growth performance (8). Qiu et al. emphasized that amino acid compositional characteristics must be thoroughly considered when utilizing cottonseed meal or low-gossypol cottonseed meal as soybean meal replacements in animal diets (9). Given the relatively lower concentrations of certain essential amino acids in cottonseed meal, which inadequately meet livestock nutritional requirements, simply increasing dietary cottonseed meal proportions without corresponding amino acid supplementation may compromise animal growth performance (10, 11). Recent evidence demonstrates that amino acid balancing, particularly through rumen-protected amino acid supplementation, can significantly improve nitrogen utilization efficiency and modulate hindgut microbiota composition in ruminants, suggesting that strategic amino acid supplementation could mitigate the limitations of alternative protein sources (12). Therefore, when selecting alternative feed ingredients to replace soybean meal, comprehensive evaluation of gross energy, protein content, and amino acid profiles is essential, with scientifically calculated substitution ratios and amino acid balancing strategies ensuring comprehensive nutritional adequacy and maintaining animal production performance.

Despite the extensive research on cottonseed meal applications in various livestock species, three critical knowledge gaps remain unaddressed. First, while cottonseed meal has been studied in aquatic species, poultry, and swine (13–15), its effects on fattening-phase crossbred sheep, particularly the economically important Dorper × Hu breed, remain largely unexplored. Second, previous studies have often overlooked the importance of amino acid balancing when substituting cottonseed meal for soybean meal, potentially confounding the interpretation of performance outcomes. Third, the comprehensive impacts of amino acid-balanced cottonseed meal diets on the complex interactions among nutrient digestibility, rumen fermentation patterns, and microbial ecology in sheep have not been systematically evaluated.

The lack of this integrated knowledge presents a significant barrier to the adoption of cottonseed meal as a sustainable protein alternative in sheep production systems, particularly in regions like Xinjiang where cottonseed meal is abundantly available but underutilized. Without clear evidence of its safety and efficacy under optimized nutritional conditions, producers remain hesitant to implement cottonseed meal-based feeding strategies, perpetuating dependence on increasingly scarce and expensive soybean meal.

Therefore, this experiment was designed to comprehensively investigate the effects of amino acid-balanced cottonseed meal substitution for soybean meal on multiple physiological and production parameters in fattening-phase Dorper × Hu crossbred sheep. By employing a systematic approach that evaluates growth performance, blood biochemical parameters, apparent digestibility, antioxidant functionality, and ruminal microbial diversity under controlled amino acid-balanced conditions, this study aims to establish optimal substitution strategies that maintain or enhance production efficiency while reducing reliance on soybean meal. The findings will provide crucial scientific evidence and practical guidelines for implementing sustainable protein feeding strategies in sheep production systems, with particular relevance to cotton-producing regions globally.

## 2 Materials and methods

### 2.1 Experimental design

The experiment was conducted at the Taikun Group Ruminant Research and Development Center. A total of 112 healthy fattening-phase Dorper × Hu crossbred rams with similar body weights (initial body weight: 22.88 ± 0.12 kg, mean ± SE) at 90 days old were selected. These sheep were randomly assigned to 4 groups, with 28 sheep in each group. The experimental duration comprised 63 days, including a 14-day adaptation period and a 49-day formal experimental period. The experimental design is presented in Table 1.

**Table 1:** Experimental diets.

Group	Dietary treatment
CON	Corn-soybean meal basal diet
T1	Cottonseed meal replacing 50% soybean meal with lysine and methionine supplementation
T2	Cottonseed meal replacing 100% soybean meal with lysine and methionine supplementation
T3	Degossypolized cottonseed meal replacing 100% soybean meal with lysine and methionine supplementation

### 2.2 Experimental diets

The experimental diets were formulated by the Taikun Group Feed Technology Center, following the nutritional requirements for Dorper × Hu crossbred sheep regarding digestible energy, crude protein, calcium, phosphorus, amino acids, mineral elements, and vitamins. Four experimental diets were formulated to be isoenergetic and isonitrogenous by design, though analyzed nutritional levels showed minor variations. To ensure the diets were iso-aminoacidic, crystalline L-lysine HCl and DL-methionine were added to the T1, T2, and T3 diets to match the calculated lysine and methionine levels of the CON diet.(dietary composition and nutritional levels are detailed in Table 2). Manufacturing was conducted by Taikun Group Changji Feed Co., Ltd.

**Table 2:** Dietary composition and nutritional levels (air-dry basis, %).

Ingredients	CON	T1	T2	T3
Corn	33.20	31.40	30.3	30.30
Wheat flour	4.50	4.50	4.50	4.50
Soybean meal	13.30	5.90	–	–
Cottonseed meal	–	6.00	10.90	–
Degossypolized cottonseed protein	–	–	–	10.90
Corn germ meal	8.00	8.00	8.00	8.00
Corn bran	6.30	9.40	11.40	11.40
Sprayed corn bran	10.00	10.00	10.00	10.00
Corn stover	20.00	20.00	20.00	20.00
Limestone	2.54	2.53	2.51	2.51
Sodium bicarbonate	0.40	0.40	0.40	0.40
Plant essential oils	0.06	0.06	0.06	0.06
Vitamin premix^1^	0.03	0.03	0.03	0.03
Trace mineral premix^2^	0.10	0.10	0.10	0.10
L-lysine HCl (78.8%)	–	0.06	0.15	0.15
DL-methionine (99%)	–	0.01	0.02	0.02
Mixed additives	1.57	1.52	1.63	1.63
Total	100.00	100.00	100.00	100.00
Nutritional levels^3^				
Digestible energy (MJ/kg)	10.80	10.80	10.80	10.80
Crude protein (%)	14.05	14.80	14.59	13.98
Rumen degradable protein (%)	8.80	8.70	8.60	8.60
Rumen undegradable protein (%)	5.70	5.80	5.90	5.90
Neutral detergent fiber (%)	35.23	32.61	33.05	34.93
Acid detergent fiber (%)	15.43	13.87	14.7	14.63
Calcium (%)	1.07	1.07	1.09	1.08
Total phosphorus (%)	0.41	0.4	0.44	0.42
Lysine (%)	0.61	0.60	0.60	0.60
Methionine (%)	0.20	0.20	0.20	0.20

^1^Vitamin supplement per kg diet: VA: 20,000 IU, VD: 5,000 IU, VE: 150 IU, niacin: 300 mg, pantothenic acid: 95 mg. ^2^Trace mineral supplement (mg/kg): Fe: 60, Mn: 45, Zn: 30, Cu: 18, I: 0.4, Se: 0.4, Co: 0.3. ^3^Crude protein, neutral detergent fiber, acid detergent fiber, calcium, and total phosphorus represent analyzed values; digestible energy, rumen degradable protein, rumen undegradable protein, lysine, and methionine represent calculated values.

### 2.3 Feeding management

Comprehensive disinfection of experimental facilities was conducted prior to trial commencement. Experimental sheep underwent routine immunization and deworming protocols. Throughout the experimental period, daily management procedures (feeding protocols, water provision, immunization schedules) were executed according to standardized operational procedures of the research facility. All animals had ad libitum access to feed and water. Daily feed offered and orts were recorded with their dry matter contents. Experimental facilities were cleaned at scheduled intervals daily while maintaining dry, well-ventilated conditions, ensuring consistent housing conditions across all treatment groups.

### 2.4 Growth performance

Individual body weights of Dorper × Hu crossbred sheep were recorded at trial initiation and termination. Feed withdrawal commenced at 21:00 on the evening preceding weighing, with measurements conducted at 09:00 the following morning, ensuring a 12-h fasting period. Dry Matter Intake (DMI), average daily gain (ADG), and feed conversion ratio (FCR) were calculated based on body weight and feed consumption data. DMI (g/d) was calculated from the difference in dry matter between offered feed and orts, and total intake and gain were used to compute FCR (DM-basis) = DMI/ADG.

DMI (g/d) = [*Σ*(Offered as-fed × DM%_offered) − Σ(Orts as-fed × DM%_orts)]/days. DM of offered diets and orts were determined (e.g., GB/T 6435–2014/AOAC 934.01).

ADG (g/d) = [Final body weight (g) − Initial body weight (g)]/Experimental days (d).

FCR = Total dry matter intake during the trial (g)/Total weight gain during trial period (g) = DMI/ADG.

### 2.5 Blood biochemical parameters

During the final week of the experimental period, six sheep were randomly selected from each treatment group for blood sampling. Collected blood samples were placed in collection tubes and allowed to stand for 30 min before centrifugation (3,000 rpm, 10 min) for serum collection. An automated biochemistry analyzer (Mindray BS-420 Automatic Biochemistry Analyzer, China) was utilized to determine alanine aminotransferase (ALT), aspartate aminotransferase (AST), alkaline phosphatase (ALP), total protein (TP), albumin (ALB), globulin (GLB), urea (UREA), lactate dehydrogenase (LDH), and creatine kinase (CK) concentrations.

### 2.6 Apparent digestibility

Fecal samples were collected during the final week preceding trial termination and preserved at −20 °C, subsequently submitted to the Taikun Changji Feed Analysis Center for expedited determination of crude protein, ether extract, neutral detergent fiber, and acid detergent fiber concentrations, facilitating nutrient digestibility calculations. Quantification of crude protein, ether extract (EE), neutral detergent fiber (NDF), and acid detergent fiber (ADF) in dietary and fecal samples was conducted in accordance with Chinese National Standards GB/T 6435–2014, GB/T 6433–2006, GB/T 20806–2006, and GB/T 20805–2006, respectively. Selected analytical methodologies referenced established protocols from “Feed Analysis and Feed Quality Testing Technology.”

Apparent nutrient digestibility (%) = [1 − (Nutrient content in feces/Nutrient content in diet) × (Acid-insoluble ash content in diet/Acid-insoluble ash content in feces)] × 100%.

### 2.7 Antioxidant indices

Serum collection protocols and preservation methodologies are detailed in section 2.5. Determination of serum total antioxidant capacity (TAOC), superoxide dismutase activity (SOD), glutathione peroxidase activity (GSH-Px), and malondialdehyde content (MDA) was performed utilizing commercial assay kits (Beijing Huaying Biotechnology Research Institute). Optical density measurements were executed using a Huawei Delang DR-200BS microplate analyzer (Wuxi Huawei Delang Instrument Co., Ltd.).

### 2.8 Ruminal short-chain fatty acids

At the end of the experiment, six representative samples from each treatment group underwent systematic slaughter procedures. Following slaughter, ruminal supernatant collection was achieved through quadruple-layer gauze filtration, with subsequent transfer to EP tubes and preservation at 4 °C pending analysis. One milliliter of ruminal supernatant was diluted with ultrapure water at a 1:2.5 ratio, followed by centrifugation at 12,000 rpm for 15 min. Subsequently, 1 mL of centrifuged supernatant was combined with 0.2 mL of 25% metaphosphoric acid solution, immediately homogenized, and incubated on ice for 30 min. Post-incubation centrifugation at 12,000 rpm for 15 min preceded collection of 0.6 mL supernatant, which was supplemented with 20 μL crotonic acid solution, homogenized, and filtered through 0.22 μm membranes. Utilizing 4-methylpentanoic acid as internal standard, volatile fatty acid (VFA) concentrations encompassing acetic acid, propionic acid, isobutyric acid, butyric acid, isovaleric acid, and valeric acid were quantified via Shimadzu GC2010 gas chromatography, with subsequent calculation of acetic to propionic acid ratios.

### 2.9 Ruminal microbial diversity

Ruminal fluid collection protocols followed methodologies delineated in Section 2.8, with filtered supernatants immediately cryopreserved in liquid nitrogen. Three representative samples per treatment group were submitted to Beijing Biomarker Technologies Co., Ltd. for comprehensive ruminal microbial diversity analysis. Total genomic DNA was extracted from ruminal contents using the TGuide S96 Magnetic Stool DNA Kit (Tiangen Biotech (Beijing) Co., Ltd.) according to manufacturer’s instructions. The hypervariable region V3-V4 of the bacterial 16S rRNA gene was amplified with primer pairs 338F: 5′-ACTCCTACGGGAGGCAGCA-3′ and 806R: 5′-GGACTACHVGGGTWTCTAAT-3′. PCR products were checked on agarose gel and purified through the Omega DNA purification kit (Omega Inc., Norcross, GA, United States). The purified PCR products were collected, and the paired-end sequencing (2 × 250 bp) was performed on the Illumina Novaseq 6,000 platform. Through systematic reads assembly and filtration, clustering or denoising procedures, followed by taxonomic annotation and abundance profiling, sample microbial community composition was elucidated.

### 2.10 Statistical analysis

Experimental data were analyzed using SPSS 26.0 statistical software. Data normality and homogeneity of variance were tested prior to analysis. Inter-group differences were evaluated using one-way analysis of variance (ANOVA) with Duncan’s multiple range test for post-hoc comparisons. Data are presented as mean ± standard error. Statistical significance was defined as *p* < 0.05, with *p* < 0.01 indicating highly significant differences.

Ruminal microbial diversity analyses were conducted on the Beijing Biomarker Technologies cloud-based analytical platform. Alpha diversity indices were compared using one-way ANOVA. Beta diversity was visualized through principal component analysis (PCA) with Adonis testing for significance. Linear discriminant analysis effect size (LEfSe) identified microbial biomarkers. PICRUSt2 software was used for functional prediction analysis. Spearman correlation analysis evaluated relationships between microbial genera and fermentation parameters.

## 3 Results

### 3.1 Effects of cottonseed meal substitution for soybean meal on growth performance parameters in Dorper × Hu crossbred sheep

As delineated in Table 3, comprehensive analysis revealed no statistically significant differences (*p* > 0.05) among the four experimental cohorts. Specifically, average daily gain (ADG) ranged from 302.50 to 353.57 g/d, and the feed conversion ratio (FCR) ranged from 4.90 to 5.63.

**Table 3:** Effects of cottonseed meal substitution for soybean meal on growth performance parameters in Dorper × Hu crossbred sheep.

Item	CON	T1	T2	T3	*P* **-value**
Initial BW, kg	22.80 ± 0.30	22.68 ± 0.14	22.73 ± 0.28	23.29 ± 0.19	0.904
Terminal BW, kg	38.43 ± 0.60	40.36 ± 0.34	37.86 ± 0.46	39.33 ± 0.58	0.577
ADG, g/d	312.50 ± 6.16	353.57 ± 6.08	302.50 ± 4.15	322.86 ± 7.72	0.183
DMI, g/d	1727.60 ± 22.22	1728.24 ± 19.85	1697.33 ± 22.88	1760.19 ± 14.53	0.907
FCR	5.59 ± 0.16	4.90 ± 0.05	5.63 ± 0.10	5.53 ± 0.19	0.424

BW, body weight; ADG, average daily gain; DMI, dry matter intake; FCR (DM-basis), DMI/ADG. Values are presented as mean ± SE.

### 3.2 Effects of cottonseed meal substitution for soybean meal on apparent digestibility in Dorper × Hu crossbred sheep

Table 4 delineates the impact of varying cottonseed meal substitution levels on nutrient apparent digestibility parameters in Dorper × Hu crossbred sheep. The findings demonstrate that cottonseed meal substitution for soybean meal exerted statistically significant effects on digestibility coefficients across all nutritional components examined (*p* < 0.05).

**Table 4:** Effects of cottonseed meal substitution for soybean meal on apparent digestibility in Dorper × Hu crossbred sheep.

Item	CON	T1	T2	T3	*P* **-value**
CP (%)	64.93 ± 0.87^b^	63.67 ± 0.20^b^	69.38 ± 0.85^a^	60.32 ± 0.53^c^	<0.001
OM (%)	62.71 ± 0.69^b^	62.65 ± 0.47^b^	61.73 ± 0.71^b^	65.25 ± 0.61^a^	0.010
EE (%)	59.10 ± 1.25^d^	68.55 ± 0.19^c^	77.44 ± 0.43^a^	71.71 ± 0.31^b^	<0.001
NDF (%)	36.64 ± 1.29^b^	32.89 ± 0.60^b^	42.88 ± 1.01^a^	27.97 ± 0.89^c^	<0.001
ADF (%)	32.80 ± 1.09^b^	27.38 ± 0.67^c^	40.01 ± 0.66^a^	24.72 ± 1.13^d^	<0.001
DM (%)	64.42 ± 0.84^b^	66.17 ± 0.20^a^	67.06 ± 0.87^a^	64.39 ± 0.36^b^	0.021

CP, crude protein; OM, organic matter; EE, ether extract; NDF, neutral detergent fiber; ADF, acid detergent fiber; DM, dry matter. Values are presented as mean ± SE. ^a,b,c,d^ Means within rows with different letter superscripts differ (*P* < 0.05).

Regarding crude protein digestibility, the T2 group exhibited the highest value (69.38%), which was significantly greater than the CON group (64.93%; *p* < 0.05). Conversely, the T3 group showed the lowest crude protein digestibility (60.32%), which was also significantly different from other groups (*p* < 0.05).

Organic matter digestibility analysis revealed that the T3 group achieved 65.25 ± 1.49%, significantly exceeding the other three treatment groups (*p* < 0.05).

Ether extract digestibility demonstrated significant inter-group variations (*p* < 0.05). The T2 group manifested the highest ether extract digestibility, followed by T3, T1, with the CON group exhibiting the lowest value. All inter-group differences achieved statistical significance.

Concerning fiber digestibility parameters, neutral detergent fiber (NDF) digestibility was maximized in the T2 group, significantly surpassing all other treatment groups (*p* < 0.05). The NDF digestibility of the T3 group was the lowest, showing a significant decrease compared to that of the other groups (*p* < 0.05).

Acid detergent fiber (ADF) digestibility similarly peaked in the T2 group, demonstrating statistical superiority over all other cohorts (*p* < 0.05). The T1 group exhibited significantly lower ADF digestibility compared to both CON and T2 groups (*p* < 0.05).

Dry matter digestibility was significantly enhanced in both T1 and T2 groups compared to the control and T3 groups (*p* < 0.05), with no difference between T1 and T2 or between CON and T3.

### 3.3 Effects of cottonseed meal substitution for soybean meal on blood biochemical parameters in Dorper × Hu crossbred sheep

Serum biochemical analysis revealed that cottonseed meal substitution for soybean meal under amino acid-balanced conditions did not significantly affect any measured blood parameters in fattening Dorper × Hu crossbred sheep (Table 5). All protein metabolism indicators, including total protein, albumin, globulin, and albumin-to-globulin ratio, remained stable across treatment groups (*p* > 0.05), indicating that cottonseed meal substitution did not disrupt protein metabolic homeostasis.

**Table 5:** Effects of cottonseed meal substitution for soybean meal on serum biochemical parameters in sheep.

Item	CON	T1	T2	T3	*P* **-value**
TP (g/L)	65.85 ± 0.99	65.78 ± 0.36	67.74 ± 0.98	66.32 ± 0.82	0.852
ALB (g/L)	33.27 ± 0.626	34.26 ± 0.59	33.29 ± 0.31	33.20 ± 0.39	0.856
GLB (g/L)	32.58 ± 0.69	31.52 ± 0.62	34.45 ± 1.06	33.13 ± 0.70	0.753
UREA (mmol/L)	9.73 ± 0.28	8.65 ± 0.10	9.03 ± 0.21	8.12 ± 0.11	0.144
AST (U/L)	69.95 ± 0.34	70.96 ± 0.49	72.38 ± 0.64	69.37 ± 0.46	0.338
ALT (U/L)	19.28 ± 0.23	20.75 ± 0.82	23.31 ± 0.82	19.05 ± 0.76	0.362
ALP (U/L)	293.95 ± 15.53	357.86 ± 21.98	310.42 ± 10.68	364.47 ± 10.14	0.463
LDH (U/L)	350.66 ± 3.24	361.23 ± 1.60	377.04 ± 4.02	371.87 ± 2.27	0.080
CK (U/L)	136.32 ± 4.14	227.88 ± 20.64	179.78 ± 3.40	165.96 ± 9.60	0.080

TP, total protein; ALB, albumin; GLB, globulin; UREA, urea; AST, aspartate aminotransferase; ALT, alanine aminotransferase; ALP, alkaline phosphatase; LDH, lactate dehydrogenase; CK, creatine kinase. Values are presented as mean ± SE.

Hepatic function enzymes (AST, ALT, and ALP) showed no significant differences among groups (*p* > 0.05), suggesting that the anti-nutritional factors in cottonseed meal, particularly gossypol, were effectively managed within the ruminal environment without inducing hepatic stress. Although serum urea concentrations exhibited a decreasing trend from the control group to the degossypolized cottonseed meal group (T3), and both LDH and CK activities showed numerical variations among treatments, these differences did not reach statistical significance (*p* = 0.14 and *p* = 0.08, respectively).

### 3.4 Effects of cottonseed meal substitution for soybean meal on antioxidant indices in Dorper × Hu crossbred sheep

**Figure 1:**
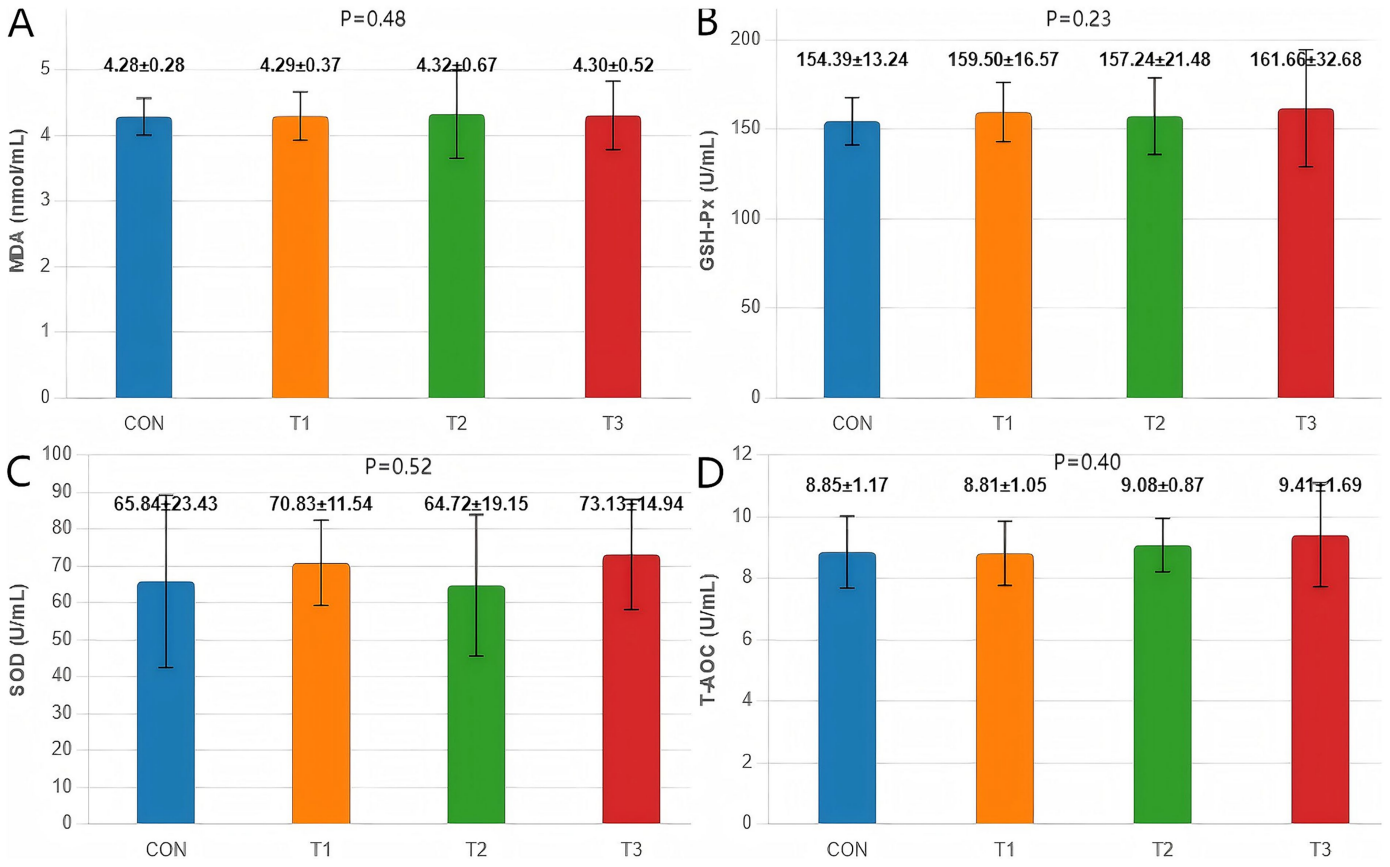
Effects of cottonseed meal substitution for soybean meal on antioxidant indices in Dorper × Hu crossbred sheep. **(A)** MDA content. **(B)** GSH-Px activity. **(C)** SOD activity. **(D)** TAOC. CON represents the control group; T1 represents 50% soybean meal replacement with cottonseed meal; T2 represents 100% soybean meal replacement with cottonseed meal; T3 represents 100% soybean meal replacement with degossypolized cottonseed meal.

Cottonseed meal substitution for soybean meal under amino acid-balanced conditions did not significantly affect any Antioxidant Indices in fattening Dorper × Hu crossbred sheep (Figure 1). Malondialdehyde content, glutathione peroxidase activity, superoxide dismutase activity, and total antioxidant capacity all remained comparable across treatment groups (*p* > 0.05).

Although the degossypolized cottonseed meal group (T3) showed numerical improvements in antioxidant enzyme activities compared to other groups, these trends did not reach statistical significance. These findings indicate that cottonseed meal substitution, whether regular or degossypolized, maintains normal antioxidant status in Dorper × Hu crossbred sheep when amino acid balance is ensured.

### 3.5 Effects of cottonseed meal substitution for soybean meal on ruminal fermentation parameters in Dorper × Hu crossbred sheep

The effects of cottonseed meal substitution for soybean meal on major volatile fatty acid (VFA) concentrations in the rumen of Dorper × Hu crossbred sheep are presented in Table 6.

**Table 6:** Effects of cottonseed meal substitution for soybean meal on ruminal fermentation parameters in Dorper × Hu crossbred sheep.

Item	CON	T1	T2	T3	*P* **-value**
Acetic acid (mmol/L)	25.75 ± 1.65	31.31 ± 3.50	27.66 ± 2.07	27.83 ± 1.55	0.420
Propionic acid (mmol/L)	9.49 ± 1.09^B^	24.03 ± 2.08^A^	18.47 ± 1.60^A^	17.99 ± 1.53^A^	<0.001
Isobutyric acid (mmol/L)	0.22 ± 0.01^A^	0.14 ± 0.01^B^	0.20 ± 0.05^A^	0.15 ± 0.01^B^	<0.001
Butyric acid (mmol/L)	3.46 ± 0.52^Bb^	11.40 ± 1.32^A^	7.64 ± 1.28^ABa^	9.15 ± 0.75^A^	<0.001
Isovaleric acid (mmol/L)	1.57 ± 0.23^A^	0.20 ± 0.02^B^	0.34 ± 0.06^B^	0.23 ± 0.03^B^	0.001
Valeric acid (mmol/L)	0.58 ± 0.05^B^	2.46 ± 0.11^A^	1.63 ± 0.27^B^	1.88 ± 0.29^B^	0.002
Acetic acid/propionic acid	2.71 ± 0.31^a^	1.30 ± 0.25^b^	1.50 ± 0.29^b^	155 ± 0.26^b^	0.028

^A,B^Means within rows with different letter superscripts differ (*P* < 0.01). ^a, b^ Means within rows with different letter superscripts differ (*P* < 0.05). Absence of superscripts or identical lowercase letter superscripts indicate non-significant differences (*P* > 0.05). Values are presented as mean ± SE.

The findings demonstrated no significant differences in ruminal acetic acid concentrations among experimental groups (*p* > 0.05). However, propionic acid concentrations in all cottonseed meal substitution groups were extremely significantly higher than in the control (CON) group (*p* < 0.01). For instance, the T1 group exhibited the highest concentration at 24.03 mmoL/L, compared to 9.49 mmoL/L in the CON group. Consequently, the acetic acid/propionic acid ratio in the CON group (2.71) was significantly higher than in the treatment groups (e.g., T1 at 1.30; *p* < 0.05).

Regarding branched-chain volatile fatty acids, the Isovaleric acid concentration in the CON group (1.57 mmol/L) was significantly higher than all cottonseed meal substitution groups (*p* < 0.01). For isobutyric acid, concentrations in the CON and T2 groups were significantly higher than those in the T1 and T3 groups (*p* < 0.001).

Furthermore, significant differences were observed in butyric acid and valeric acid concentrations among groups. The butyric acid concentration in the CON group was significantly lower than the T1 and T3 groups (*p* < 0.01), and significantly lower than the T2 group (*p* < 0.05). For valeric acid, the T1 group concentration was significantly higher than all other groups (*p* < 0.01).

### 3.6 Effects of cottonseed meal substitution for soybean meal on ruminal microbiota in Dorper × Hu crossbred sheep

#### 3.6.1 Analysis of ruminal microbial *α*-diversity

**Figure 2:**
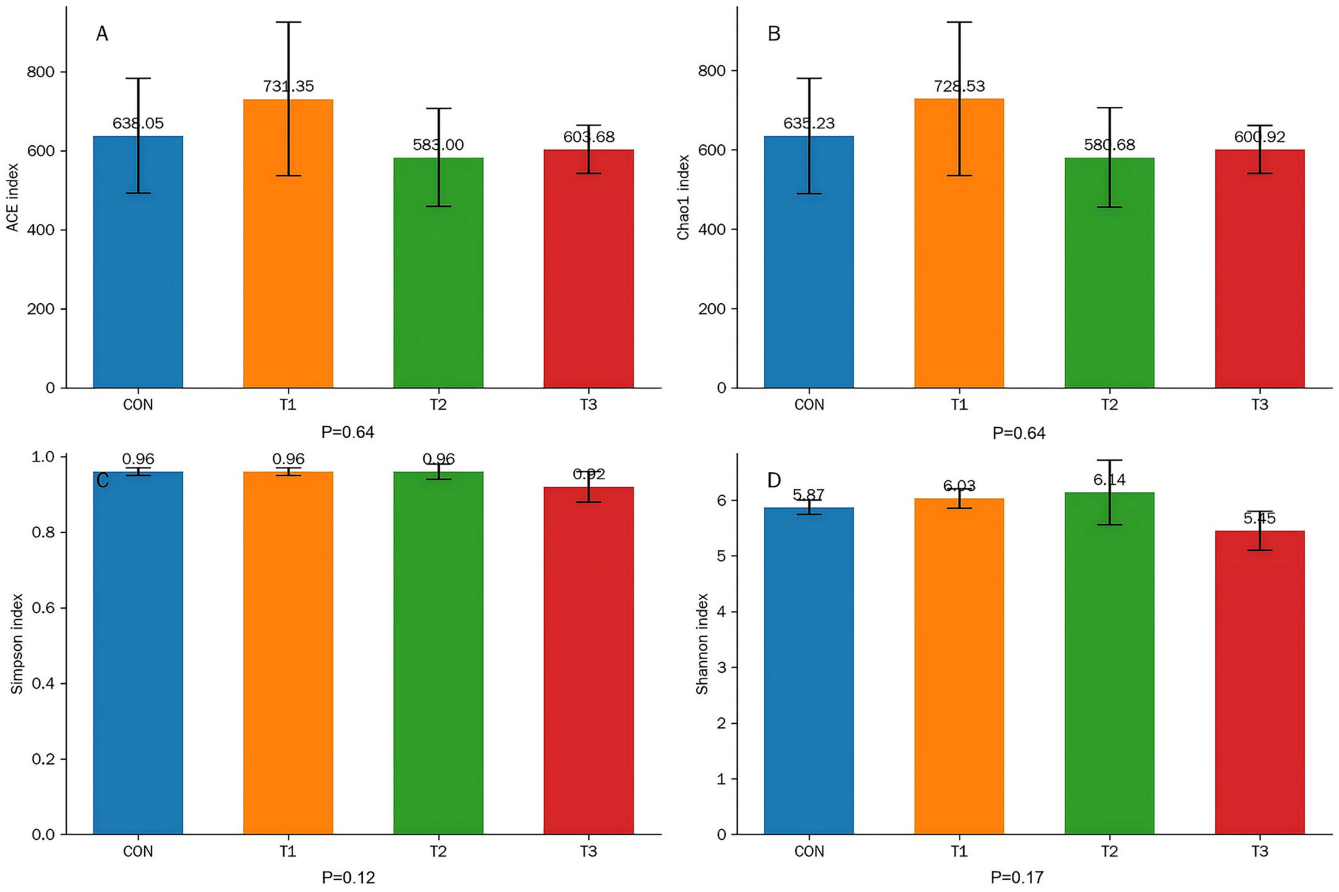
Effects of cottonseed meal substitution for soybean meal on ruminal microbial *α*-diversity in Dorper × Hu crossbred sheep. **(A)** ACE index. **(B)** Chao1 index. **(C)** Shannon index. **(D)** Simpson index.

To evaluate the effects of different dietary treatments on the richness and diversity of bacterial communities in the rumen of Dorper × Hu crossbred sheep, α-diversity indices were calculated, with results presented in Figure 2.

Data analysis revealed no significant differences among treatment groups (CON, T1, T2, T3) in ACE and Chao1 indices (reflecting community richness) or Shannon and Simpson indices (comprehensively reflecting community diversity) (*p* > 0.05). Specifically, *p*-values for both ACE and Chao1 indices were 0.64, while *p*-values for Shannon and Simpson indices were 0.17 and 0.12, respectively.

These results indicate that under amino acid-balanced conditions, substitution of soybean meal with varying proportions of cottonseed meal or degossypolized cottonseed meal did not produce significant adverse effects on the overall richness and diversity of ruminal microbial communities in fattening Dorper × Hu crossbred sheep, suggesting that the ruminal microecosystem maintained relative stability across different dietary treatments.

#### 3.6.2 Analysis of ruminal microbial *β*-diversity

**Figure 3:**
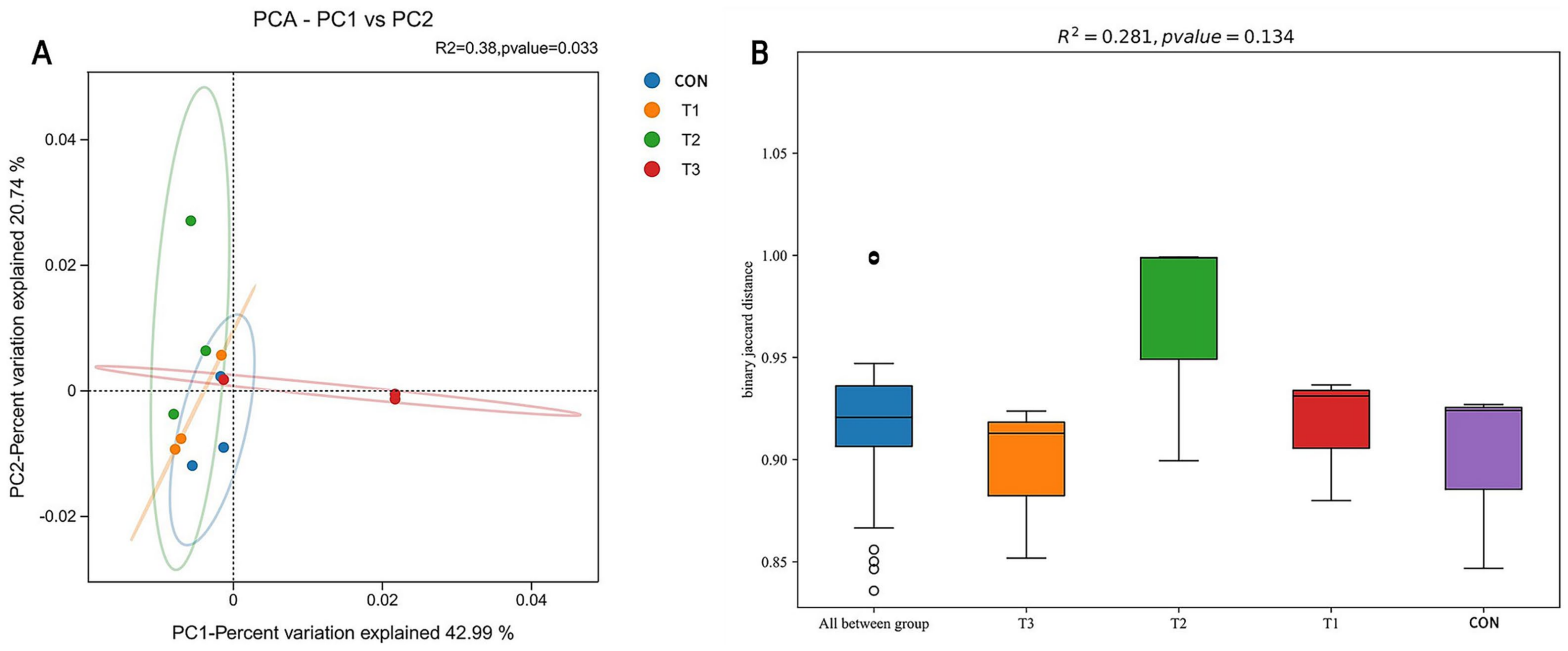
*β*-diversity analysis of ruminal microbial communities in Dorper × Hu crossbred sheep. **(A)** Principal Component Analysis (PCA) plot of ruminal microbial communities. **(B)** Inter-group difference analysis of β-diversity based on Jaccard distance. The PCA plot **(A)** displays the distribution of samples from different treatment groups in terms of microbial community structure, with different colored points representing different treatment groups; closer points indicate more similar community structures. The Jaccard distance box plot **(B)** illustrates the degree of dissimilarity among samples from each group.

To evaluate the effects of different cottonseed meal substitution levels on ruminal microbial community structure in Dorper × Hu crossbred sheep, β-diversity analysis was performed. Principal Component Analysis (PCA) results are presented in Figure 3A, where scattered points represent samples from different treatment groups, with inter-point distances reflecting the degree of difference in microbial community composition between samples. The visualization demonstrates that sample points from the CON group (control), T1 group (50% soybean meal replaced with cottonseed meal), T2 group (100% soybean meal replaced with cottonseed meal), and T3 group (100% soybean meal replaced with degossypolized cottonseed meal) did not form distinctly independent clusters in the two-dimensional ordination plot. This indicates that the ruminal microbial community structures of Dorper × Hu crossbred sheep across different cottonseed meal substitution levels were generally similar, without significant separation.

Further analysis of inter-group significance using Adonis (permutational multivariate analysis of variance based on distance matrices) revealed no significant differences in microbial community structure among treatment groups (*p* > 0.05). As illustrated in Figure 3B, *β*-diversity analysis based on Jaccard distance yielded similar conclusions, with substantial overlap in box plots among groups, indicating non-significant differences in microbial community composition between groups. These findings demonstrate that under amino acid-balanced conditions, complete substitution of soybean meal with cottonseed meal or degossypolized cottonseed meal did not exert substantial effects on the ruminal microbial community structure of Dorper × Hu crossbred sheep during the late fattening period.

#### 3.6.3 Taxonomic annotation and differential analysis of ruminal microbial community composition

**Figure 4:**
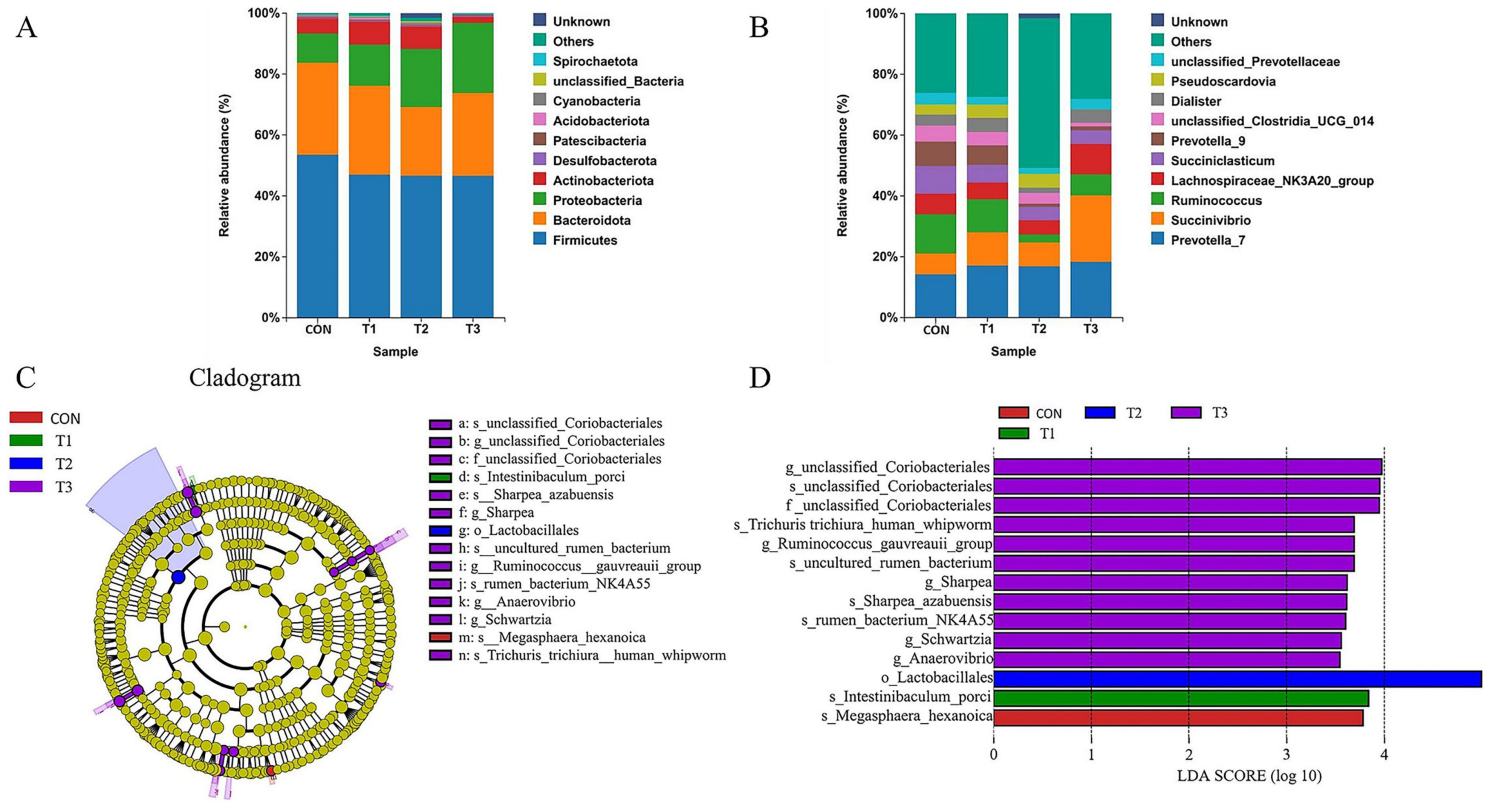
Taxonomic annotation and differential analysis of ruminal microbial community composition. **(A)** Relative abundance bar chart at phylum level. **(B)** Relative abundance bar chart at genus level. **(C)** Cladogram. **(D)** Linear discriminant analysis diagram. **(C)** Represents the control group; T1 represents 50% soybean meal replacement with cottonseed meal; T2 represents 100% soybean meal replacement with cottonseed meal; T3 represents 100% soybean meal replacement with degossypolized cottonseed meal.

To comprehensively investigate the effects of different dietary treatments on ruminal microbial community structure in Dorper × Hu crossbred sheep, 16S rRNA gene sequencing analysis was performed on samples from each group, with results presented in Figure 4.

At the phylum taxonomic level (Figure 4A), ruminal microbial communities across all treatment groups were predominantly composed of Firmicutes and Bacteroidota, representing core microbiota involved in fiber degradation and vitamin B synthesis within the rumen. Additionally, Proteobacteria, Actinobacteriota, Desulfobacterota, Verrucomicrobiota, Campylobacterota, Patescibacteria, and Spirochaetota were present at relatively lower abundances across groups. No significant differences in microbial composition at the phylum level were observed among groups (*p* > 0.05), indicating that cottonseed meal substitution for soybean meal did not significantly impact the macrostructure of ruminal microbial communities.

At the genus taxonomic level (Figure 4B), Lachnospiraceae_NK3A20_group, Prevotella_7, Succinivibrio, and Succiniclasticum emerged as predominant genera across all groups. Prevotella plays a pivotal role in plant polysaccharide degradation and propionic acid production, while Succinivibrio and Succiniclasticum are associated with succinate-to-propionic acid conversion, critical for host energy metabolism. Lachnospiraceae primarily participates in butyric acid production. Furthermore, unclassified_Prevotellaceae, Prevotella_9, unclassified_Clostridia_UCG_014, Pseudoscardovia, and Dialister were present at varying abundances.

To identify microbial taxa with significant abundance differences under different dietary treatments, Linear discriminant analysis Effect Size (LEfSe) analysis was conducted (Figures 4C,D). Results revealed 14 microbial biomarkers exhibiting significant differences among groups. Specifically: Control group (CON): Enriched exclusively with Megasphaera_hexanoica. T1 group (50% soybean meal replacement with cottonseed meal): Significantly enriched with Intestinibaculum_porci. T2 group (100% soybean meal replacement with cottonseed meal): Characterized by significant enrichment of the order Lactobacillales (o_Lactobacillales) as a biomarker. T3 group (100% soybean meal replacement with degossypolized cottonseed meal): Exhibited the greatest microbial enrichment diversity, comprising 11 taxa including g_unclassified_Coriobacteriales, Ruminococcus_gauvreauii_group, f_unclassified_Coriobacteriales, Trichuris_trichiura_human_whipworm, g_Sharpea, Schwartzia, Anaerovibrio, s_Sharpea_azabuensis, s_uncultured_rumen_bacterium, s_unclassified_Coriobacteriales, and s_rumen_bacterium_NK4A55.

#### 3.6.4 Functional prediction of ruminal microbiota

**Figure 5:**
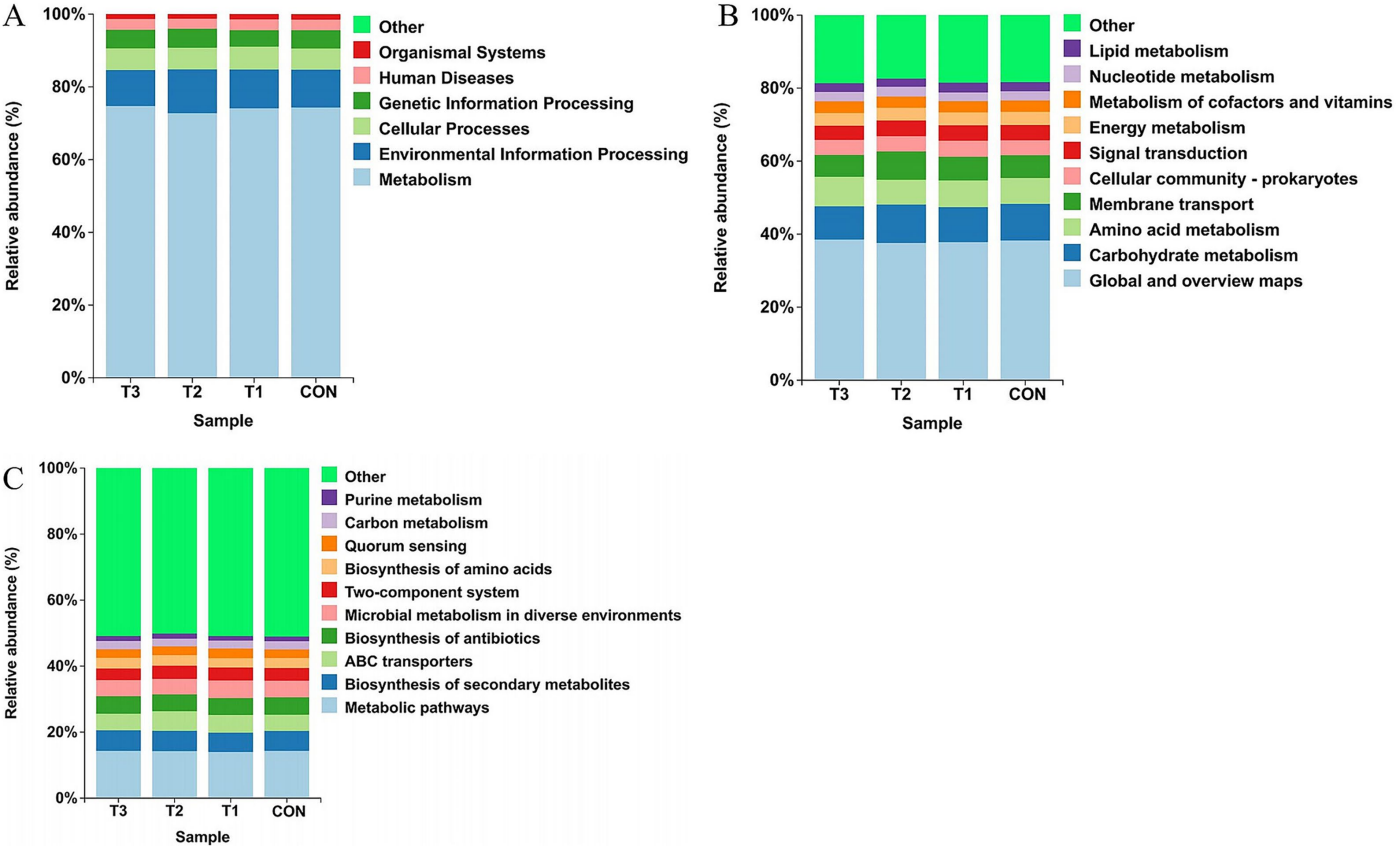
Functional prediction of ruminal microbiota. Differential analysis of KEGG metabolic pathways at **(A)** Level 1, **(B)** Level 2, and **(C)** Level 3.

To investigate the effects of different dietary treatments on the functional potential of ruminal microbial communities in Dorper × Hu crossbred sheep, this study conducted predictive functional analysis using the Kyoto Encyclopedia of Genes and Genomes (KEGG) database based on 16S rRNA sequencing data, with results presented in Figure 5.

At the KEGG Level 1 functional classification (Figure 5A), ruminal microbial functions across all treatment groups were predominantly enriched in “Metabolism,” with relative abundances averaging over 70% across groups, representing the absolute dominant functional pathway. This was followed by “Environmental Information Processing” and “Cellular Processes.” Distribution patterns of relative abundances for major functional categories remained highly consistent among treatment groups, with no significant differences observed.

At KEGG Level 2 (Figure 5B), subdivision of metabolic pathways revealed that “Global and overview maps,” “Carbohydrate metabolism,” “Amino acid metabolism,” and “Membrane transport” constituted the most abundant sub-functional categories. Consistent with Level 1 findings, these functional pathways maintained remarkable stability in distribution between the control group (C) and cottonseed meal substitution groups (T1, T2, T3), with no apparent fluctuations in relative abundances.

In the refined analysis at KEGG Level 3 (Figure 5C), “Metabolic pathways,” “Biosynthesis of secondary metabolites,” “ABC transporters,” and “Two-component system” emerged as principal predicted pathways. Despite more specific functional classification, ruminal microbial functional profiles remained stable across dietary treatment groups, demonstrating no significant differences.

#### 3.6.5 Correlation analysis between major ruminal genera and fermentation parameters and functional pathways

**Figure 6:**
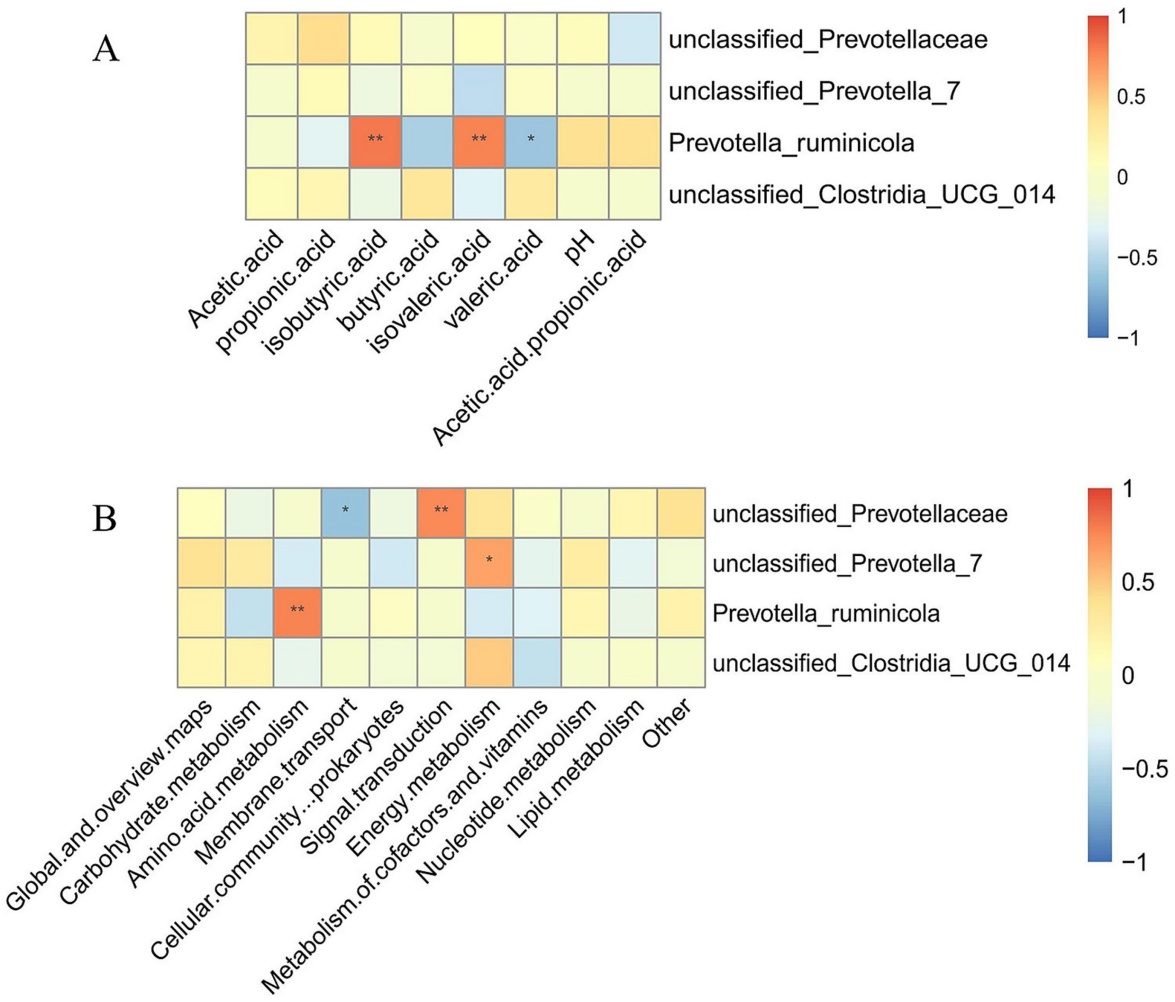
Correlation analysis between major ruminal genera and fermentation parameters and functional pathways (genus level). **(A)** Correlation analysis between ruminal microbial composition and ruminal fermentation parameters. **(B)** Correlation analysis between ruminal microbial composition and microbial community functions.

To further elucidate the interrelationships between ruminal microbiota and fermentation products as well as community functions, this study performed Spearman correlation analysis between the top 10 most abundant microbial genera and ruminal short-chain fatty acid (VFA) concentrations and KEGG functional pathway relative abundances (Figure 6).

Regarding ruminal fermentation parameters (Figure 6A), results demonstrated that the relative abundance of *Prevotella ruminicola* exhibited highly significant positive correlations with isobutyric acid and isovaleric acid concentrations (*p* < 0.01), while showing a significant negative correlation with valeric acid concentration (*p* < 0.05).

Concerning microbial community functions (Figure 6B), the relative abundance of unclassified_Prevotellaceae demonstrated a highly significant positive correlation with Signal transduction function (*p* < 0.01), while exhibiting a significant negative correlation with Membrane transport function (*p* < 0.05). The relative abundance of unclassified_Prevotella_7 showed a significant positive correlation with Energy metabolism function (*p* < 0.05). Furthermore, the relative abundance of *Prevotella ruminicola* demonstrated a highly significant positive correlation with Amino acid metabolism function (*p* < 0.01).

## 4 Discussion

### 4.1 Effects of cottonseed meal substitution for soybean meal on growth performance parameters in Dorper × Hu crossbred sheep

The present investigation demonstrates that under amino acid-balanced conditions, substitution of soybean meal with cottonseed meal or degossypolized cottonseed meal (at 50% or 100% replacement rates) exerted no significant effects on growth performance parameters in fattening Dorper × Hu crossbred sheep (*p* > 0.05). These findings indicate that cottonseed meal maintains growth performance comparable to control groups when essential amino acids are balanced through lysine and methionine supplementation, mitigating potential limitations from cottonseed meal’s relatively lower essential amino acid content (9).

Our findings align with existing ruminant studies. Wanapat et al. (1) demonstrated that cottonseed meal in concentrate produced no negative effects on feed intake or nutrient digestibility in young dairy bulls, supporting cottonseed meal’s potential as a sustainable protein alternative. Cao et al. reported no significant differences in growth performance among fattening sheep fed corn-cottonseed meal diets supplemented with amino acids (16). Similarly, Yang et al. (10) demonstrated that 30–50% cottonseed meal substitution in Hu sheep maintained feed conversion ratios between 5.0–5.6, closely aligning with our findings (4.9–5.63).

The efficacy of cottonseed meal varies by species due to digestive physiology differences. Ruminants utilize cottonseed meal effectively when diets are properly balanced (17), largely because ruminal microbiota can detoxify anti-nutritional factors. Wang et al. (18) isolated a rumen Lactobacillus strain capable of degrading over 80% of free gossypol, underscoring the ruminant’s natural detoxification capacity. Consistent with this, our T3 group (degossypolized cottonseed meal) showed no advantage over T2 (regular cottonseed meal), suggesting effective ruminal gossypol detoxification. The 14-day adaptation period allowed adequate acclimation to cottonseed meal diets.

These findings provide positive evidence supporting cottonseed meal application in Dorper × Hu crossbred sheep fattening diets, particularly relevant for alleviating soybean meal shortages in major cotton-producing regions (15). Future investigations should explore higher substitution rates and long-term feeding effects to optimize economic benefits and sustainability.

### 4.2 Effects of cottonseed meal substitution for soybean meal on apparent digestibility in Dorper × Hu crossbred sheep

The T2 group (100% cottonseed meal replacement with amino acid balance) demonstrated significantly enhanced digestibility for crude protein (69.38%), ether extract (77.44%), NDF (42.88%), and ADF (40.01%) compared to controls (*p* < 0.05). These improvements contrast with previous reports where cottonseed meal supplementation reduced nutrient digestibility in sheep (19, 20). The divergence likely stems from our amino acid balancing strategy, which mitigated anti-nutritional factor impacts while the higher fiber content in cottonseed meal potentially stimulated ruminal microbial activity and fiber-degrading enzyme secretion (21).

The superior digestibility in amino acid-balanced diets reflects the critical role of balanced amino acid profiles in optimizing protein biological value and nutrient utilization efficiency in ruminants. This balancing appears to ameliorate the negative impacts of cottonseed meal anti-nutritional factors through enhanced protein synthesis efficiency and improved ruminal microbial function.

Unexpectedly, the T3 group (degossypolized cottonseed meal) exhibited significantly lower crude protein and fiber digestibility than T2, despite gossypol removal. This paradox likely resulted from the degossypolization process causing excessive protein denaturation through solvent extraction or thermal treatment, forming complexes resistant to ruminal degradation (22). However, T3’s highest organic matter digestibility suggests enhanced utilization of non-protein, non-fiber organic fractions. These contrasting results warrant further investigation into how processing methods affect cottonseed meal protein structure and digestibility.

### 4.3 Effects of cottonseed meal substitution for soybean meal on blood biochemical indices in Dorper × Hu crossbred sheep

Blood biochemical indices constitute critical parameters for evaluating nutritional status, metabolic function, and health conditions in animals (23). Under amino acid-balanced conditions, substitution of soybean meal with cottonseed meal or degossypolized cottonseed meal (at 50% or 100% replacement rates) exerted no significant effects on serum biochemical indices in fattening Dorper × Hu crossbred sheep (*p* > 0.05).

The stability of protein metabolism indicators—including total protein, albumin, globulin, and albumin-to-globulin ratio—across all treatment groups indicates that cottonseed meal substitution did not disrupt protein metabolic homeostasis. This outcome likely resulted from the isonitrogenous dietary design coupled with strategic amino acid supplementation, which ensured adequate nitrogen utilization while mitigating potential limitations from the relatively lower essential amino acid content in cottonseed meal (9, 24).

Hepatic function enzymes (AST, ALT, and ALP) showed no significant inter-group differences, suggesting that anti-nutritional factors from cottonseed meal, particularly gossypol, were effectively degraded within the ruminal environment without inducing detectable hepatic damage. Although serum urea concentrations and muscle enzyme activities (LDH and CK) exhibited numerical trends approaching significance (*p* < 0.10), all values remained within normal physiological ranges, potentially reflecting subtle modulation of nitrogen and energy metabolism.

These findings align with ruminant studies demonstrating the safety of cottonseed meal as a soybean meal substitute. Gil-Miranda et al. (25) observed that complete cottonseed meal substitution in growing lambs maintained stable protein metabolism indicators despite affecting immunoglobulin levels. Similarly, Zhao et al. (19) reported that gossypol supplementation in Duolang sheep did not significantly alter serum enzyme levels due to effective ruminal detoxification. This contrasts markedly with monogastric animals, which lack ruminal detoxification capacity and often exhibit elevated hepatic enzymes when fed high-gossypol cottonseed products.

The methodological rigor employed—including iso-energetic and iso-nitrogenous diet formulation with amino acid balancing—was essential for isolating the specific effects of protein source substitution while minimizing confounding variables (26). This approach ensures that the observed biochemical stability can be confidently attributed to the successful integration of cottonseed meal rather than compensatory nutritional adjustments.

### 4.4 Effects of cottonseed meal substitution for soybean meal on antioxidant indices in Dorper × Hu crossbred sheep

Under amino acid-balanced conditions, cottonseed meal substitution for soybean meal did not significantly affect antioxidant indices in fattening Dorper × Hu crossbred sheep (*p* > 0.05). Malondialdehyde content, glutathione peroxidase activity, superoxide dismutase activity, and total antioxidant capacity remained comparable across all treatment groups. Although not statistically significant, the T3 group (100% degossypolized cottonseed meal) exhibited numerical improvements in antioxidant enzyme activities, potentially attributable to the degossypolization process reducing free gossypol content while preserving beneficial bioactive compounds with antioxidant properties (27).

The stability of antioxidant parameters aligns with findings in ruminants demonstrating cottonseed meal’s favorable safety profile. Rehemujiang et al. (21) reported that fermented cottonseed/rapeseed meal replacement in Hu sheep maintained normal antioxidant enzyme activities. The consistently low MDA levels (4.28–4.32 nmol/mL) across groups indicate that cottonseed meal substitution did not induce lipid peroxidation, which has important implications for meat quality maintenance, as emphasized by Ponnampalam et al. (28) regarding the direct influence of feed antioxidants on meat oxidative stability.

The mechanistic role of amino acid balance in maintaining antioxidant stability likely involves methionine serving as a glutathione synthesis precursor, supporting GSH-Px activity (29), and balanced amino acids promoting antioxidant enzyme synthesis through improved protein synthesis efficiency (30). Wang et al. (31) investigated the effects of oat supplementation on Small-tailed Han sheep in 2023 and found that alterations in amino acid profiles were closely associated with meat quality, indicating complex interactions between amino acid metabolism and antioxidant systems. Additionally, the plant essential oils included in all experimental diets (0.06%) may have contributed to antioxidant parameter stability through direct free radical scavenging and upregulation of endogenous antioxidant enzyme expression (32), creating synergistic effects with balanced amino acids to maintain robust antioxidant defense across all cottonseed meal substitution levels.

### 4.5 Effects of cottonseed meal substitution for soybean meal on ruminal fermentation parameters in Dorper × Hu crossbred sheep

Ruminal volatile fatty acids (VFAs) constitute the primary energy source for ruminants, accounting for approximately 70–80% of their metabolizable energy requirements (33). Under amino acid-balanced conditions, cottonseed meal substitution for soybean meal significantly altered ruminal fermentation patterns in fattening Dorper × Hu crossbred sheep.

The most notable effect was on propionate production. Ruminal propionic acid concentrations in all cottonseed meal substitution groups (T1, T2, T3) were highly significantly elevated compared to the control group (*p* < 0.01), with T1 showing the highest concentration at 24.03 mmol/L versus 9.49 mmol/L in controls. This finding aligns with Ponnampalam et al. (28) who reported enhanced total ruminal VFA production with fermented cottonseed meal substitution. Since propionate serves as the principal gluconeogenic precursor in ruminants, providing 60–74% of carbon for glucose synthesis (34), the increased propionic acid production in cottonseed meal groups suggests improved energy metabolism efficiency.

Consequently, the acetic acid-to-propionic acid ratio was significantly lower in all cottonseed meal substitution groups (1.30–1.65) compared to the control group (2.71; *p* < 0.05). Lower acetate-to-propionate ratios are typically associated with enhanced energy utilization efficiency (35). These findings align with Yusuf et al. (36) who observed increased molar proportions of acetate and propionate with fermented TMR containing cottonseed meal.

Butyric acid concentrations were significantly higher in cottonseed meal substitution groups than controls (*p* < 0.01), potentially reflecting adaptive adjustments in ruminal microbial community structure (33). This aligns with Ma et al. (37) who found that enzymatically hydrolyzed cottonseed peptides enhanced both acetic acid and butyric acid content. Similarly, Wang et al. (38) found that cottonseed byproducts could enhance ruminal fermentation efficiency through augmented lipid and amino acid metabolism.

Branched-chain volatile fatty acids showed distinct patterns. Isovaleric acid concentration in the CON group (1.57 mmol/L) was highly significantly elevated compared to all cottonseed meal substitution groups (*p* < 0.01). Since branched-chain VFAs primarily originate from branched-chain amino acid deamination (39), lower concentrations in cottonseed meal groups likely reflect the relatively lower branched-chain amino acid content in cottonseed meal.

The improved fermentation patterns observed with cottonseed meal substitution, particularly enhanced propionate production and reduced acetate-to-propionate ratios, contrast with *in vitro* findings by Wang et al. (40) showing inhibitory effects of high gossypol levels on ruminal fermentation. This discrepancy may be attributed to: (1) the degradation capacity of ruminal microorganisms against gossypol, reflecting microbial community resilience to anti-nutritional compounds (41); (2) the amino acid balancing strategy potentially mitigating negative effects; and (3) the use of degossypolized cottonseed meal in the T3 group.

These results demonstrate that cottonseed meal substitution combined with amino acid balancing not only maintains normal ruminal fermentation but may improve energy utilization efficiency through enhanced propionic acid production. The potential for further optimization exists through synergistic interactions with functional additives, as suggested by Romero et al. (42) who found that appropriate hydrogen acceptors could increase acetate and total VFA production when methane production is inhibited. This has significant implications for practical production applications in regions where cottonseed meal is abundantly available.

### 4.6 Effects of cottonseed meal substitution for soybean meal on ruminal microbiota in Dorper × Hu crossbred sheep

The rumen microbiome plays a crucial role in feed conversion and animal production performance (43). This study evaluated the effects of cottonseed meal substitution on ruminal microbial diversity through 16S rRNA sequencing, revealing that the ruminal microecosystem maintained relative stability under different protein source substitutions.

Alpha diversity indices (ACE, Chao1, Shannon, Simpson) showed no significant differences among treatment groups (*p* > 0.05), consistent with Wang et al. (38) who found similar results when feeding cottonseed hulls to sheep. This stability may be attributed to the amino acid balancing strategy ensuring nutritional equilibrium and the strong homeostatic regulatory capacity of the ruminal microecosystem (44). Similarly, Zhou et al. (45) reported minimal effects of enzymatically hydrolyzed cottonseed protein on bacterial structure and composition.

Although *β*-diversity analysis showed overall similar microbial community structures, LEfSe analysis revealed distinct microbial enrichment patterns. Notably, the T3 group (degossypolized cottonseed meal) enriched the most microbial species (11 taxa), including Coriobacteriales, Sharpea, Schwartzia, and Anaerovibrio. This aligns with Wang et al. (46) who found that cottonseed byproduct components significantly correlated with attached bacterial composition, suggesting degossypolization may create favorable microenvironments for specific microbial growth.

At the phylum level, Firmicutes and Bacteroidota remained dominant across all groups, maintaining their role as core microbiota for ruminal fiber degradation. At the genus level, Prevotella maintained its dominant position, potentially explaining increased propionic acid production in cottonseed meal groups. Succinivibrio and Succiniclasticum, involved in succinate-to-propionate conversion, remained stable, contributing to enhanced propionate production (47).

Correlation analysis revealed significant relationships between microbial composition and fermentation parameters. *Prevotella ruminicola* showed highly significant positive correlations with isobutyric and isovaleric acid concentrations (*p* < 0.01), consistent with Ma et al. (37) who demonstrated that cottonseed peptides could improve ruminal fermentation by modulating specific microbial taxa.

KEGG functional prediction showed high stability at the functional pathway level despite compositional differences. Metabolism-related functions accounted for over 70%, with consistent distribution of key pathways across groups, demonstrating functional redundancy that ensures fermentation function maintenance despite feed composition changes (48). The correlation between unclassified_Prevotellaceae and signal transduction function (p < 0.01) suggests cottonseed meal may regulate fermentation through microbial signaling processes (49).

The enrichment of characteristic microbes in the T3 group may reflect substrate property changes from degossypolization treatment. Wang et al. (18) isolated *Lactobacillus agilis* WWK129 with 83% gossypol degradation capability, highlighting the potential for specific microbes in gossypol metabolism. These findings demonstrate that under amino acid-balanced conditions, cottonseed meal substitution maintains ruminal microbial homeostasis while modulating specific taxa associated with improved fermentation efficiency.

## 5 Conclusion

This study concludes that cottonseed meal, when balanced for essential amino acids, can fully replace soybean meal in the diets of fattening crossbred sheep without impairing growth performance. The primary scientific contribution is the demonstration that this substitution confers significant metabolic advantages, evidenced by enhanced apparent digestibility of crude protein, fiber, and fat. Furthermore, the observed increase in ruminal propionate concentration suggests improved energy utilization efficiency, establishing cottonseed meal as not only a viable but a metabolically superior alternative protein source. These findings provide a strong nutritional basis for utilizing cottonseed meal to develop more economically resilient and sustainable ruminant production systems.

Future research should prioritize three areas: (1) investigating how different degossypolization methods affect protein structure to elucidate their impact on nutrient digestibility; (2) conducting long-term trials to assess the cumulative effects on final meat quality and reproductive performance; and (3) performing comprehensive economic analyses to validate the cost-effectiveness of this feeding strategy under diverse market conditions.

## Data Availability

The original contributions presented in the study are publicly available. This data can be found here: https://www.ncbi.nlm.nih.gov/bioproject/PRJNA1347654, accession number: PRJNA1347654.
